# Novel Drug Delivery Systems Tailored for Improved Administration of Glucocorticoids

**DOI:** 10.3390/ijms18091836

**Published:** 2017-08-24

**Authors:** Fred Lühder, Holger M. Reichardt

**Affiliations:** 1 Institute of Neuroimmunology and Institute for Multiple Sclerosis Research, University Medical Centre Goettingen, 37075 Göttingen, Germany; 2 Institute for Cellular and Molecular Immunology, University Medical Center Goettingen, 37073 Göttingen, Germany

**Keywords:** glucocorticoids, nanoparticles, drug delivery systems, neuroinflammation, rheumatoid arthristis, cancer, liposomes

## Abstract

Glucocorticoids (GC) are one of the most popular and versatile classes of drugs available to treat chronic inflammation and cancer, but side effects and resistance constrain their use. To overcome these hurdles, which are often related to the uniform tissue distribution of free GC and their short half-life in biological fluids, new delivery vehicles have been developed including PEGylated liposomes, polymeric micelles, polymer-drug conjugates, inorganic scaffolds, and hybrid nanoparticles. While each of these nanoformulations has individual drawbacks, they are often superior to free GC in many aspects including therapeutic efficacy when tested in cell culture or animal models. Successful application of nanomedicines has been demonstrated in various models of neuroinflammatory diseases, cancer, rheumatoid arthritis, and several other disorders. Moreover, investigations using human cells and first clinical trials raise the hope that the new delivery vehicles may have the potential to make GC therapies more tolerable, specific and efficient in the future.

## 1. Introduction

Glucocorticoids (GC) are amongst the oldest synthetic drugs used in modern medicine. After their initial purification from the adrenal gland by Edward C. Kendall in the 1930s, it also became possible to synthesize these compounds chemically in larger quantities, resulting in the first application of cortisone to rheumatoid arthritis (RA) patients in the late 1940s [1,2]. Since then, numerous synthetic GC with different chemical modifications and biopharmaceutical properties have been developed. They are administered orally, intravenously, or topically, and phosphate or acetate derivatives are often employed to increase their solubility in aqueous solution [3]. The major characteristic of GC are their strong anti-inflammatory and immunosuppressive effects, which makes it possible to use them in the treatment of a variety of inflammatory conditions such as RA, multiple sclerosis (MS), asthma, graft-versus-host disease, lymphoma, or skin inflammation [4]. Notwithstanding the powerful therapeutic effects of GC, they can also cause serious side effects, in particular when administered at high doses and/or after long-term use. Thus, it is very important to determine whether there is a reasonable benefit-to-risk ratio [5]. Frequently observed adverse effects comprise osteoporosis, hypertension, muscle wasting, hyperglycemia, edema formation, gastrointestinal disturbances, growth retardation and mood changes [6,7,8,9]. In addition, GC resistance is encountered in many patients and considerably constrains therapeutic success [10]. Consequently, there is a continuous need to improve this therapeutic regimen. Earlier approaches to address this issue include the development of more selective GC analogs [11,12] and nitrosteroids [13], or a combination of GC with dipyridamole [14]. More recently, chronotherapeutic GC formulations that enable a restricted release of the drug were approved by the FDA [15,16]. Despite undisputable improvements, side effects remain a serious concern. Therefore, research efforts aimed at the development of better pharmaceutical formats are still warranted.

In recent years, a number of innovative drug delivery systems have been developed which aim at targeting specific tissues or cell types and attempt to achieve a timely controlled drug release resulting in stable serum levels over a prolonged time [17]. Another goal of using carrier systems is to reduce degradation and clearance of applied compounds, leading to improved bioavailability and pharmacokinetics. Drug delivery systems that fulfill these criteria include liposomes and nanoparticles (NP), as well as inorganic scaffolds. Nanosize materials further have the advantage that, due to their small size, they are specifically taken up through gaps in the lining endothelial cells, for instance in inflamed tissue. In this review, we focus on currently available organic and inorganic carrier systems employed for the application of GC with a specific emphasis on their use as anti-inflammatory agents in diseases of the central nervous system (CNS). We summarize findings made in experimental animal models, elude on their cellular and molecular mechanisms of action, and provide an outlook on their potential clinical application.

## 2. Mechanisms of GC Action

GC are steroid hormones that are naturally produced by the adrenal gland and released in response to stimuli such as physical stress. Due to their hydrophobic nature, GC passively cross the cell membrane and bind to the GC receptor (GR), which resides in the cytosol as part of a multimeric protein complex. Subsequently, this complex breaks apart and the bound GR translocates into the nucleus where it regulates gene expression or, in rare cases, remains in the cytosol to impact signaling pathways [18,19]. In recent years, a number of transcriptional mechanisms have been described by which the GR controls transcription [20]. When first discovered, the GR was thought to mainly activate gene expression by homodimeric binding to GC response elements (GRE) present in regulatory regions of many genes [21,22]. Since then, several other mechanisms have been described by which the GR represses gene expression. GR dimers and monomers can bind to negative GRE, inverted repeat GRE, or GRE in close vicinity to nuclear factor κβ (NF-κβ) and activator protein 1 (AP-1) binding sites, and thereby inhibit transcription [23]. In addition, tethering of the monomeric GR to other transcription factors without DNA-binding can result in repressive activity [24]. Thus, the GR is able to downregulate gene expression by multiple mechanisms.

For many years, it was believed that beneficial effects could be separated from adverse ones by restricting GC activity to the repressive mechanism mediated by GR monomers. For this reason, considerable efforts have been made to identify drugs with such features, so-called selective GR agonists (SEGRA). This approach led to the discovery of ZK216348 and Compound A, which efficiently ameliorate skin inflammation and experimental autoimmune encephalomyelitis (EAE) in mice [25,26]. Further support for this concept came from the analysis of mice expressing a GR with a defective dimerization interface (GR^dim^ mice) [27]. Notably, EAE induced in these mice remains fully responsive to GC therapy [28], suggesting that GR dimerization may not be required for the anti-inflammatory activity of GC. In contrast, GR^dim^ mice are prone to develop systemic inflammation after challenge with lipopolysaccharide (LPS) or tumor necrosis factor alpha (TNFα) [22,29] and are refractory to GC treatment of RA and asthma [30,31]. These findings suggest that SEGRA may not be the optimal choice to make GC therapy more specific. Observations in conditional GR knock-out mice provided an alternative rationale for improvement as they revealed that GC modulate distinct cell types in each individual disease. For instance, T cells are essential targets for the treatment of EAE, whereas macrophages and neutrophils are critical for control of contact dermatitis by free GC [32,33]. It is against this background that new approaches aimed to deliver GC to specific cell types have gained much interest recently. The underlying concept is that drugs are no longer administered in their free form but rather covalently bound or physically attached to vesicles, macromolecules, NP, or scaffolds, which restricts their access to selected organs and cell types and results in a more controlled release. This should in turn reduce adverse effects and make the therapy more tolerable.

## 3. Drug Delivery Systems for GC Application

**Figure 1:**
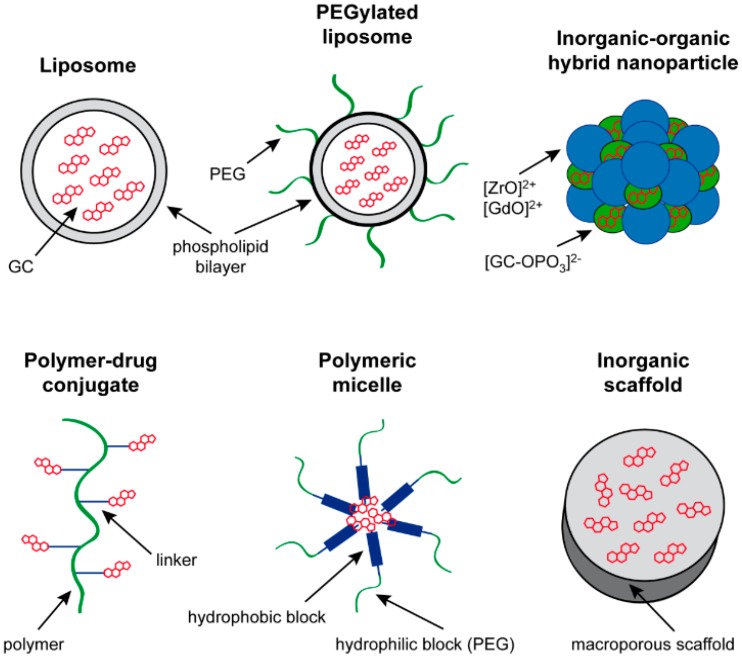
Current material concepts for GC delivery. Liposomes are vesicles composed of a phospholipid bilayer that are able to carry GC in their lumen. Modification of their surface by polyethylene glycol (PEG) results in the generation of so-called PEGylated liposomes with improved characteristics. GC are mostly encapsulated in the liposomal cavity in the form of their hydrophilic phosphate or acetate derivatives. In a polymer-drug conjugate, the GC is covalently bound to a macromolecular carrier via a linker, whereas polymeric micelles are spherical structures consisting of a hydrophilic block that can be PEG and a central hydrophobic block where the GC are contained. Besides organic vehicles, there are also inorganic material concepts such as large scaffolds to which GC can be adsorbed. Inorganic-organic hybrid nanoparticles are a new development which is composed of an inorganic cation, e.g., [ZrO]^2+^ or [GdO]^2+^, and a functional organic anion such as a phosphorylated GC with the composition [GC-OPO_3_]^2−^, both of which assemble into particles in a similar manner as sodium chloride.

GC are widely employed in the treatment of a plethora of diseases, but their use is complicated by serious adverse effects and a considerable rate of treatment failure caused by steroid-resistance. In addition, small molecular weight compounds such as GC face many challenges beyond the need of being pharmacologically active. They are hydrophobic molecules and poorly soluble in biological fluids, although this feature can be improved by phosphorylation or acetylation. Nevertheless, solubility has a considerable impact on the pharmacokinetics, for which reason administration of GC in their free form indeed results in a short biological half-life in the circulation due to rapid degradation and renal clearance. Another disadvantage of free GC is their largely uniform and unspecific distribution within the human body, which can result in side effects and limited drug availability in target tissues. Thus, it would be desirable to have delivery systems at hand that overcome these hurdles and, in particular, increase the half-life of GC and direct them to the site of action, i.e., inflamed tissues or tumor cells. It is believed that new nanoscale carrier systems could fulfill these expectations and solve some of the challenges imposed on traditional GC therapy. However, despite the recent enthusiasm for nanomaterials, they might also be potentially toxic, depending on their concentration, solubility, shape and size, so that their use can be problematic. Every type of drug formulation has its advantages and disadvantages, and this is why different delivery systems have been developed and tested over the years to identify the most suitable one for each application (Figure 1 and Table 1).

### 3.1. Liposomes

Liposomes are biocompatible vesicles composed of a phospholipid-bilayer with structural resemblance to cell membrane (Figure 1 and Table 1), which form small spheroids that are able to carry both hydrophilic and lipophilic drugs [76,77]. Unless modified, these structures are opsonized by plasma proteins resulting in a relatively rapid engulfment and clearance by the phagocytic system. Therefore, strategies have been developed to modify the surface of the liposomes to avoid immediate phagocytosis and to enhance circulation time in the blood. Rapid opsonization can be prevented by using fully saturated lipids and a high cholesterol content [78], but also by the introduction of molecules at the surface which act as a “shield” and prevent recognition by plasma proteins (Figure 1). One popular strategy is the use of polyethylene glycol (PEG), which protrudes from of the surface and ensures a substantially increased half-life [79,80]. Owing to their size, PEGylated liposomes are specifically suitable for passively targeting tumors and sites of inflammation where they accumulate due to the “enhanced permeability and retention effect” (EPR) [76,81]. In addition, active targeting can be achieved by inducing the specific uptake of the liposomes by certain cell populations, for instance through binding to E-selectin on the endothelium [82]. Other examples include liposomes that are conjugated to a CD74-specific antibody targeting B cell lymphoma [34], and liposomes modified by sialic acid-octadecylamine [35] or heparin-sodium deoxycholate [36] for specific delivery to tumor cells. In addition, a PEG derivative has been used to target synovial fibroblast-like cells [44], and liposomes modified with a CD163-specific antibody recognizing a subpopulation of anti-inflammatory macrophages have been tested in a model of Parkinson’s disease [55].

Another important aspect of liposomes is the drug release rate, which is dependent on its composition, the nature of the drug and the method of encapsulation. It could be shown that liposomes containing prednisolone (PDN) have a favorable near-zero-order kinetic of drug release with almost no initial peak, ensuring a constant administration of the compound over a long time period [83]. Although liposomes are generally inert and thus few adverse effects and toxicity were expected, immunogenicity to PEG after repeated injection has still been reported [84,85]. In addition, an activation of the complement system has been observed that could constrain application of PEGylated liposomes to patients [86]. Nevertheless, liposomes in different forms remain the most investigated drug delivery carrier systems for GC in both preclinical studies and clinical trials.

### 3.2. Polymeric Micelles

Another nanoformulation used for the delivery of GC are polymeric micelles (Figure 1 and Table 1), which are spherical, colloidal NP with a diameter of 10–100 nm [87]. They consist of a core-shell structure with a hydrophilic corona and a hydrophobic core which contains the drug. Polymeric micelles are formed by self-assembly of amphiphilic copolymers in aqueous solution. PEG is typically used as a hydrophilic block whereas the hydrophobic block may consist of poly ε-caprolactone (PCL) or poly (l-lactide). Encapsulating the hydrophobic drug in the core of the polymeric micelles allows its solubilization and protection from degradation in biological milieus. Furthermore, it results in an extended circulation time and a slower release of the compound. Similar to liposomes, the EPR effect plays an important role for the targeting of the drug to tumors and inflamed tissues. In addition, active delivery to specific cell types can also be achieved for this nanoformulation by attaching antibodies, sugar moieties or cell-penetrating peptides. It is noteworthy that the complement system was found to be activated to a lesser extent by polymeric micelles than by liposomes, thus presumably reducing nanomaterial-related side effects [66]. Taken together, polymeric micelles have the potential to improve GC delivery although cell-type specific approaches have not yet been reported.

### 3.3. Polymer-Drug Conjugates

Pharmacologically active compounds can be covalently bound to macromolecular carriers by using linkers, which results in conjugates that are stable in blood but labile in an inflammatory environment, tumor tissues or certain intracellular compartments [88]. Using this strategy, long-circulating delivery systems can be obtained that are suitable for both passive and active drug targeting (Figure 1 and Table 1). The most popular example of this class of drug carriers are *N*-(2-hydroxypropyl) methacrylamide (HPMA) copolymers, which have been used to conjugate various small drugs, for instance dexamethasone (Dex), doxorubicin or gemcitabine [67,89]. To achieve well-controlled drug loading and low polydispersity, the active compound is introduced into the monomers prior to polymerization. When covalently bound to the copolymer via a pH sensitive linker, the drug becomes cleaved in an acidic environment [67]. Alternative strategies for drug release are the use of redox, enzyme or light sensitive linkers [90]. HPMA copolymers have been shown to be non-immunogenic, but possible toxic effects of the polymer have not been studied in detail.

Due to their low molecular weight, small size and hydrophobicity, application of anti-cancer and anti-inflammatory agents in their free form is often complicated by rapid renal clearance, hepatic degradation, and the tendency to accumulate in healthy tissues rather than reaching tumors or areas of inflammation. In contrast, polymer-drug conjugates have an increased physical stability and a prolonged retention time in the circulation. In addition, a fraction of the material is taken up by blood leukocytes and thus actively transported to the site of action. While passive drug targeting using HPMA copolymers is widely believed to depend on the EPR effect [88,91], some authors argue that the ELVIS mechanism (Extravasation through Leaky Vasculature and Inflammatory cell-mediated Sequestration) might better explain the mode of action of these carriers, at least in the case of local and systemic inflammatory conditions [92]. Interestingly, the molecular weight and the drug content were found to impact the half-life and clearance of polymer-drug conjugates but not the rate of internalization by macrophages [92]. Another advantage of the nanoformulation at hand over the free drug is its multimodality. An HPMA copolymer containing doxorubicin and gemcitabine for instance was successfully used to simultanously target two anti-cancer drugs to tumors [89]. Furthermore, a polymer–Dex conjugate containing a near-infrared or a fluorescent dye could be used to determine its distribution in vivo by imaging analysis [92]. Polymer-Dex conjugates have also been evaluated for their capacity to circumvent GC side effects that often accompany the treatment of inflammatory diseases. In fact, induction of osteolysis, a disadvantage of GC therapy, was reduced when employing such a macromolecular drug carrier, while the anti-inflammatory activity of GC was retained [71]. This confirms that the specificity of GC treatment can be increased using such an approach. A disadvantage of HPMA copolymers is, however, the non-biodegradable nature of the polymer backbone, thus stimulating further research for improved material concepts [93].

### 3.4. Inorganic Drug Delivery Systems

Not all drug delivery systems are based on organic molecules but they may also consist of different types of inorganic material. Such vehicles can be designed as large transplantable scaffolds or systemically administered NP (Figure 1 and Table 1). A macroporous polydimethylsiloxane (PDMS) scaffold for instance has been tested for the delivery of GC in a mouse model of diabetes [94]. Small discs made from PDMS were used as a 3-D platform for transplantation of islets and simultaneous local delivery of Dex, which led to a significantly increased grafting efficacy in mice.

GC can be attached not only to large scaffolds but also to inorganic NP, one example being the use of clay minerals such as laponite (LAP), which form transparent colloidal dispersions in water [95]. Hydrophobic GC can be deposited on the surface through hydrogen bonding [72], and since LAP is transparent, it is suitable for intraocular injection, enabling a slow drug release in the eye [72]. Another example is hydroxyapatite, a nanosized ceramic [74]. GC can attach to the surface of such carriers, resulting in the formation of small NP. Collectively, inorganic carriers may be useful for the delivery of GC in selected application, although a highly defined drug load is difficult to achieve.

### 3.5. Inorganic–Organic NP

In the case of inorganic drug carriers, the functional organic molecule is passively attached to the matrix by adsorption. Thus, the stoichiometry is difficult to control and the drug load is low. To circumvent this problem, a novel concept of inorganic–organic hybrid NP (IOH-NP) has been developed [96]. Such IOH-NP have the general composition [ZrO]^2+^[*R_function_*OPO_3_]^2−^ and represent a new platform of materials suitable for drug delivery in the treatment of multiple disorders such as inflammation, cancer and infection. Similar to sodium chloride, IOH-NP consist of equimolar amounts of an inorganic cation such as [ZrO]^2+^ or [GdO]^2+^ and a functional organic anion with a phosphate ([*R_function_*OPO_3_]^2−^) or sulfonate ([*R_function_*OSO_3_]^2−^) group (Figure 1 and Table 1). The anion can either be a pharmacologically active compound such as betamethasone phosphate [BMP]^2−^, or a fluorescent dye such as flavin mononucleotide [FMN]^2−^ or indocyanine green [ICG]^2−^ [96]. Precipitation of both compounds in aqueous solution results in the formation of NP with a hydrodynamic diameter of 30–40 nm that are insoluble in water and characterized by their high drug content that accounts for up to 90% of the total mass [75]. Due to the flexible synthesis, it is possible to combine several different anion species in one single NP. This feature allows simultaneous drug delivery and multimodal detection by fluorescent microscopy, flow cytometry, in vivo imaging in the near-infrared range, and magnetic resonance tomography (MRT) based on the paramagnetism of gadolinium. The suitability of IOH-NP to deliver GC has been confirmed both in vitro and in vivo [75]. [ZrO]^2+^[(BMP)_0.9_(FMN)_0.1_]^2−^ NP (BMP-NP) were preferentially taken up by macrophages, could be detected by fluorescent techniques, and efficiently suppressed clinical symptoms of EAE in mice. Although potentially toxic effects of the inorganic compound of the IOH-NP have not been finally excluded yet, their use as a drug delivery vehicle is very promising since they combine an extremely high drug load with very distinct cell type specificity.

## 4. GC Nanoformulations in the Treatment of Disorders of the CNS

### 4.1. Treatment of Neuroinflammatory Diseases

**Figure 2:**
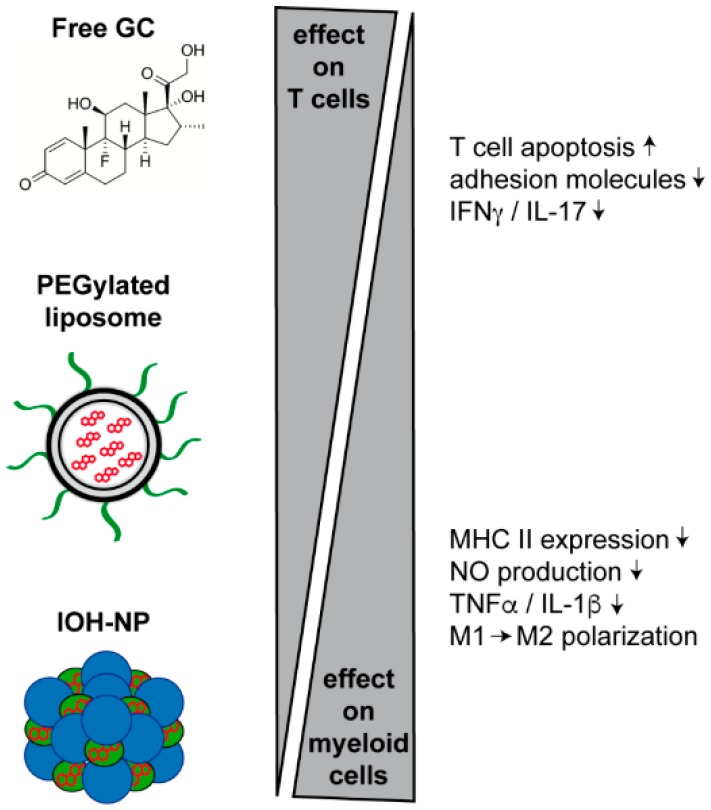
Comparison of different delivery vehicles in modulating EAE and immune cell functions in mice. Treatment of EAE in mice using free GC is mostly mediated via their effects on T cells, namely apoptosis induction and a reduction of adhesion molecules and cytokines such as IFNγ and interleukin (IL)-17. In contrast, GC delivered via PEGylated liposomes or inorganic–organic hybrid nanoparticles (IOH-NP) rather impact on myeloid cells by lowering MHC II surface levels and NO production, and inhibiting TNFα and IL-1β expression. These effects result in a shift of macrophage polarization from M1 to M2. In general, the activity of IOH-NP depends more strictly on the targeting of myeloid cells than it is the case for PEGylated liposomes.

GC are a mainstay in the treatment of acute relapses in MS patients [97]. Although they are used for only short periods and therefore cause limited side effects, the dose which is applied is very high, thus warranting more specific drug delivery systems. An initial study revealed that long-circulating PEGylated liposomes encapsulating PDN accumulated in the spinal cord and were more effective than free GC in active and passive rat EAE models in attenuating clinical symptoms and improving histopathology [56]. In a chronic rat EAE model, PEGylated liposomes containing methylprednisolone (MP) exerted more pronounced effects than those loaded with PDN [57]. Mechanistic studies were later performed in a MOG-induced EAE model using conditional GR knock-out mice [58]. While free Dex had been previously found to target T cells in EAE [32], long-circulating liposomes encapsulating PDN mostly affected myeloid cells and caused a shift of their phenotype from M1 (classically activated) to M2 (alternatively activated), corresponding to their clinical efficacy (Figure 2). Consequently, the therapeutic efficacy of this drug formulation was reduced when the GR was specifically deleted in myeloid cells and almost abrogated when the GR was absent from both T cells and myeloid cells. This observation argues that liposomes act via both cell types [58]. Surprisingly, the situation was different when BMP-NP were used for the delivery of GC [75]. Here, the therapeutic effect was completely lost when the GR was absent in myeloid cells whereas deletion of the GR in T cells did not impact therapeutic success at all [75]. Mechanistic studies revealed that GC delivered via IOH-NP also caused a switch from an M1 to an M2 phenotype similar to PEGylated liposomes (Figure 2). Taken together, different immune cells are targeted by GC that are delivered either as a free drug (almost exclusively T cells), encapsulated in PEGylated liposomes (T cells and myeloid cells) or IOH-NP (almost exclusively myeloid cells).

Several follow-up studies confirmed the clinical efficiency of GC encapsulated in PEGylated liposomes using rat and mouse EAE models without shedding further light on the mechanism [59,60]. Liposomes loaded with MP and stabilized by phosphatidylcholine were effective in the treatment of actively induced and passive transfer EAE, but attempts to specifically target the liposomes to the CNS by peptides binding to either ApoE or β-amyloid failed to improve the therapeutic benefit [61]. In some studies less T-cell infiltration and demyelination within the spinal cord was detected, consistent with an effect of GC on T-cell migration as observed previously [28,32]. Furthermore, magnetic resonance imaging (MRI) studies revealed fewer lesions in animals treated with liposomal Dex, suggesting that it may directly restore the integrity of the blood–brain barrier [61], but direct evidence for this conclusion is missing.

### 4.2. Treatment of Other CNS Diseases

GC delivered by different drug carrier systems have also been tested in other models of CNS disorders. For instance, a copolymer consisting of PEG and PCL has been used for anti-inflammatory therapy with Dex in a mouse model of spinal cord injury. The NP were superior to free Dex in terms of resolving inflammation and showed a slow and long-lasting release of the drug [70]. In a rat model of Parkinson’s disease, liposomes were designed to target CD163^+^ macrophages and deliver Dex to this cell type [55]. Some of these liposomes were found to be carried into the CNS where they presumably modulated the microglia, leading to reduced neurodegeneration and better motor performance.

GC are also used as an anti-inflammatory treatment after stroke. To test whether the use of an alternative drug delivery vehicle would enhance this effect, Dex was encapsulated in long-circulating liposomes and compared to the free drug in an experimental model in rats [62]. Application of liposomal Dex resulted in an improved behavioral outcome and, although the lesion size was not different between the groups, animals receiving Dex via liposomes showed a larger salvaged tissue area [62]. Together, GC administered by alternative drug delivery systems were successfully applied in neurological conditions with an involvement of the immune system.

## 5. Alternative GC Delivery in the Treatment of Other Diseases

### 5.1. Cancer

GC are used for two main purposes in the context of cancer therapy. First, adverse effects of chemotherapy are at least partly manageable with GC. They prevent edema formation and hypersensitivity upon administration of taxel [98] and diminish general side effects such as weight loss, fatigue, nausea and vomiting [99,100]. Second, GC enhance anti-cancer effects of some drugs and have tumoricidal potential themselves in a number of malignancies [101]. The most widespread use of GC is the treatment of leukemia and lymphoma, where predominantly their pro-apoptotic property is exploited [102,103]. Due to the nature of these diseases, GC have to be administered for a very long period, which enhances the risk of serious side effects. Therefore, a more directed delivery to malignant cells would be desirable to enhance local drug concentration and to concomitantly decrease it in other organs. Some encouraging studies in this respect have been performed in recent years, some of which we describe below.

Long-circulating liposomes encapsulating PDN, MP or Dex showed good clinical efficacy and a more sustained activity compared to the free drug in various neoplastic mouse models including melanoma, colon carcinoma, B-cell leukemia, as well as in a model mimicking prostate cancer bone metastasis [37,38,39]. Short-circulating liposomes, however, had only a minor effect, underscoring the importance of the drug formulation for clinical outcome [37]. When comparing different GC contained in long-circulating liposomes in a melanoma model, budesonide (Bud) had the strongest effect followed by Dex, whereas MP and PDN had a lower potency [40] despite comparable tumor localization [41]. In the same model, long-circulating liposomes were compared to polymeric micelles for their suitability to deliver GC [42]. Although the latter vehicle also released the drug over a period of hours to days, its clinical efficacy was slightly lower than that of liposomes [42]. To achieve active targeting, liposomes encapsulating Dex were conjugated to a CD74-specific antibody and tested on primary chronic lymphocytic leukemia cells in vitro and a xenograft model in vivo [34]. The cytotoxic and therapeutic effects were enhanced compared to both the free drug and liposomes without the antibody. A liposomal system for the co-delivery of Dex and doxorubicin was tested in vivo in a mouse sarcoma tumor model and turned out to be superior to liposomes containing only the single components [35]. In another approach, mitoxantrone and PDN were co-delivered by using liposomes conjugated to low molecular weight heparin-sodium deoxycholate to improve target binding [36]. This system showed higher efficacy in tumor inhibition in mouse models when compared to liposomal mitoxantrone and PDN alone [36]. In a theranostic approach, a multimodal NP-emulsion composed of iron oxide nanocrystals for MRI detection, a fluorescent dye and PDN was tested in a mouse colon carcinoma model and showed good accumulation in the tumor combined with therapeutic efficacy [73].

It has been postulated that locally delivered GC often act on tumor-associated macrophages (TAM), which impact angiogenesis and tumor growth [104,105,106]. Indeed, PEGylated liposomes containing PDN had a stronger anti-proliferative and anti-inflammatory effect on endothelial cells than the free drug, and especially the expression of pro-angiogenic proteins was strongly diminished [40,107]. In contrast, an oil-in-water NP-emulsion with PDN did not reduce angiogenesis but rather enhanced macrophage infiltration [73]. The notion that TAM are direct targets of liposomal GC was challenged by a report showing that therapeutic efficacy and drug accumulation within the tumor do not always correlate [43]. Furthermore, the uptake of liposomes by TAM in this study was low and did not lead to TAM depletion. This finding suggested that the anti-tumor effect was presumably mediated by other mechanisms, for instance an inhibition of monocyte infiltration [43].

In summary, GC have been successfully applied in cancer therapy in different animal models, either alone or in combination with other chemotherapeutic drugs using different delivery vehicles, in particular liposomes. While clinical efficacy was superior to the free drug in most cases, the cellular and molecular mechanisms remain poorly understood.

### 5.2. Rheumatoid Arthritis

Historically, RA is the first disorder to be successfully treated with GC [1]. Although there is a broad spectrum of disease-modifying anti-rheumatic drugs available today, GC are still used as a first-line therapy or in combinational regimens due to the fast treatment response and the efficient reduction of erosive joint damage. Current treatment guidelines recommend the use of GC in early rheumatoid arthritis patients, albeit at the lowest effective dose and for the shortest duration possible [108,109]. Hence, in this field, strong efforts are also being made to identify better drug formulations and therapeutic schemes, as summarized below.

To achieve a dose reduction and to diminish side effects, PEGylated liposomes with PDN were tested in an antigen-induced arthritis model. Similar to EAE experiments [58], liposomal drug delivery was more effective in suppressing joint inflammation than the use of the free compound [45]. In line with these results, GC delivered by other nanoformulations such as PCL-PEG micelles [66], HPMA copolymers [67], or hydroxyapatite NP [74] showed a clear clinical benefit and higher efficacy than the free drug in rat RA models, confirming their potential usefulness in anti-inflammatory therapy. Liposomal PDN suppressed bone erosion by reducing osteoclast activity, inhibiting osteoclast precursor cell differentiation [46], and selectively interfering with the M1 polarization of synovial lining macrophages [47]. Specific uptake in affected joints could be demonstrated by PET/CT, which also made it possible to monitor disease progression and drug response [48]. Clinical efficacy could be further enhanced by targeting GC containing liposomes to synovial fibroblasts and endothelial cells by attaching homing peptides [44]. Sterically stabilized liposomes were even superior to the TNFα inhibitors Infliximab and Etanercept [49]. A comparison between Dex-containing liposomes, Dex-conjugated polymeric micelles and HPMA Dex-copolymers confirmed that a slow drug release also resulted in a prolonged therapeutic activity [50].

In the case of HPMA Dex-copolymers, a favorable arthrotropism was achieved by increasing its molecular weight, although this approach also led to an enrichment of Dex in the spleen [68]. A higher influx of the conjugated Dex into the inflamed joints could be directly demonstrated by MRI [51]. Furthermore, an extensive uptake of the Dex-copolymer by synovial fibroblasts and myeloid cells was observed, accompanied by a reduced production of pro-inflammatory mediators such as cytokines and matrix metalloproteases [51].

Besides studying therapeutic efficacy, some authors also addressed GC-related side effects. PEGylated liposomes with PDN were found to have little effects on liver enzymes but still suppressed the HPA-axis [45]. Liposomal Dex effectively suppressed clinical symptoms in a rat model of RA but resulted in hyperglycemia and a loss of body weight, which was not observed when liposomal PDN and Bud was used [52]. Non-PEGylated liposomes with Dex were also superior to the free drug in terms of therapeutic efficacy, but the joint-specific localization was lost and the unspecific uptake in spleen and liver increased [53,54]. Thus, the observed clinical effects presumably result from unspecific systemic actions on immune cells outside the target organ. Finally, evaluating the skeletal toxicity using micro-computed tomography in a mouse model of RA revealed that application of HPMA Dex-copolymers resulted in reduced skeletal toxicity compared to free GC [69].

### 5.3. Application of GC Nanoformulations in Other Preclinical Studies

GC are a standard therapy of asthma. Due to the need to use this medication chronically, it was investigated whether liposomal encapsulation of Bud allows a reduction of the treatment frequency in a mouse model of allergic asthma. It turned out that weekly administration of liposomal Bud was as effective as a daily administration of the free drug [63], not only concerning the attenuation of clinical symptoms but also with regard to peripheral blood eosinophil counts, eosine peroxidase activity in the bronchoalveolar lavage and serum IgE levels [64]. In another study, PEGylated liposomes were applied in a spontaneous mouse model of systemic lupus erythematosus, where they prolonged survival and ameliorated clinical symptoms [110]. As liposomes often tend to accumulate in liver and spleen, they were also tested in the treatment of acute and chronic liver injury in mice [65]. In a chronic toxic liver fibrosis model and an acute hepatitis model, liver damage was reduced after application of Dex-containing liposomes. This observed effect was due to an induction of T-cell apoptosis and lower levels of pro-inflammatory cytokines whereas hepatic Kupffer cells were increased and adopted an M2 phenotype [65]. While these studies focused on liposomes, a polymer–Dex conjugate was tested in a mouse model of ulcerative colitis and found to result in a more long-lasting activity than the free drug and in a better efficacy to ameliorate clinical symptoms [71]. Taken together, these examples underscore the great potential of the new drug delivery systems of GC in a variety of clinical applications.

## 6. From Bench to Bedside-Transfer to the Clinic

Despite numerous encouraging preclinical studies performed with alternative systems for GC delivery and the current clinical use of liposomal formulations to deliver compounds such as doxorubicin or vincristine to patients [111], surprisingly little effort has been made to translate findings made on GC nanoformulations into the clinic. Possible reasons may include the widely held view that “liposome-mediated therapies have largely failed to increase anticancer efficacy over conventional formulations” [112]. Additionally, the hope that liposomes were largely devoid of side effects was dashed by the observation that they could activate the complement system and elicit immune responses leading to enhanced clearing after repeated application.

Up to now, several GC formulations have been investigated using human cells and tested in clinical trials. PEGylated liposomes with Dex increased the expression of pro-inflammatory cytokines by human macrophages under basal conditions but reduced them in the presence of LPS [113]. Additionally, the migration of human macrophages and monocytes was inhibited by this nanoformulation [113]. IOH-NP containing BMP were shown to modulate features of human monocytes, for instance the expression of cytokines and the migratory behavior [75]. The results of a clinical trial investigating the application of PDN-containing long-circulating liposomes in artherosclerosis therapy showed an increased half-life of the drug and its uptake by macrophages isolated from iliofemoral plaques of patients. In contrast, arterial wall permeability and inflammation were not reduced [114]. Another registered clinical trial (NCT00241982) testing the efficacy and safety of PEGylated liposomes containing PDN against free MP in the treatment of RA patients was terminated. The initial results presented in form of a meeting abstract were encouraging and showed that the liposomes were well tolerated and resulted in better improvement compared to the free drug [115], but further details were not published. Another clinical trial (NCT02534896) investigating GC nanoformulations is currently recruiting patients [116]. Taken together, translational studies are still scarce but nevertheless continue to be promising.

## 7. Conclusions

**Figure 3:**
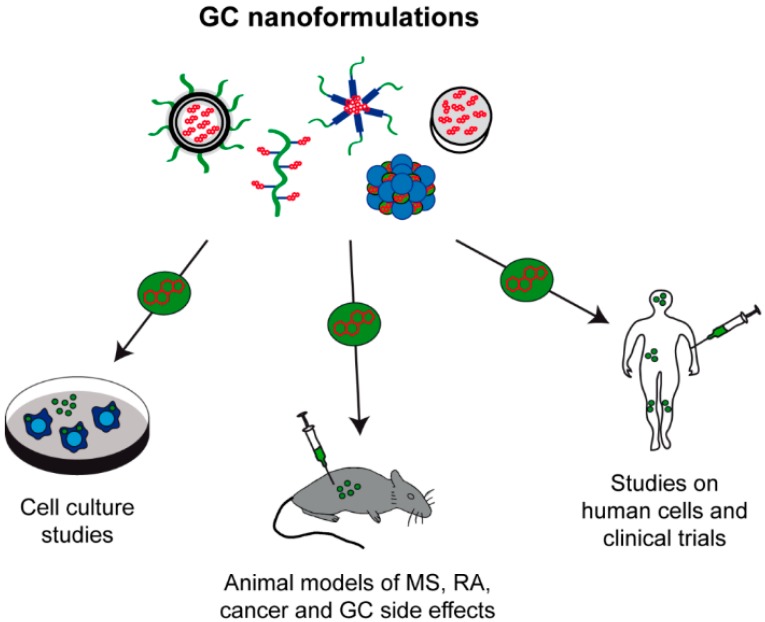
Current state of preclinical and clinical research on GC nanoformulations. Liposomes, polymeric micelles, polymer-drug conjugates, inorganic materials and IOH-NP have been intensively tested in vitro and in vivo. The majority of analyses were performed in animal models of various diseases including multiple sclerosis (MS), rheumatoid arthritis (RA), cancer and GC-related side effects, but translational studies in human cells were also started and even a few clinical trials were already initiated. However, none of the current concepts made it into clinical practice so far.

GC have been applied in medical therapy for more than half a century and have provided efficient relief of clinical symptoms to millions of patients. Nevertheless, GC therapy can also cause suffering when administration results in the development of osteoporosis, muscle wasting or growth retardation in children. For this reason, enormous research efforts have been made for many years to optimize this therapeutic regimen, e.g., by chemically modifying the drugs. More recently, approaches aimed at a more targeted delivery of GC have been initiated as it was recognized that many drawbacks were related to the uniform tissue distribution of GC and their short half-life in biological fluids. Chemists are constantly designing new delivery vehicles based on organic and inorganic materials with improved features, and biologists and medical researchers test their application in vitro and in vivo (Figure 3). Many promising results have been celebrated in the past years but the translation into the clinic remains a challenge and progress is slow. It has to be considered that delivery vehicles indeed often reduce GC-related complications but at the same time produce other adverse effects linked to the employed material. Thus, every nanoformulation has not only to be tested for its therapeutic efficacy and reduced side effects of the drug but also for the absence of material-related toxicity and immune responses against the vehicle. This is a time-consuming process which has to proceed through many steps of preclinical and clinical development, and thus it does not come as a surprise that as yet only few clinical trials have been initiated. None of the nanomedicines described in this review have made it into the clinic so far but results are mostly promising, which makes us believe that great successes are still to come. The concept of a long-lasting drug delivery to specific cell types and tissues is attractive and will presumably be the future of GC application.

## Figures and Tables

**Table 1:** Features and medical applications of different drug delivery systems used for GC application.

Carrier System	Structural Details	Concerns and Limitations	Medical Applications	References
Liposomes and PEGylated liposomes	- Small spheroids consisting of a lipid bilayer; - Modification of the surface (e.g., PEG or antibodies) to increase stability and half-life and to enhance target specificity	- Immunogenicity of PEG; - Activation of the complement system	- Cancer; - Rheumatoid arthritis; - Parkinson`s disease; - EAE; - Stroke; - Asthma; - Liver injury	[34,35,36,37,38,39,40,41,42,43]; [44,45,46,47,48,49,50,51,52,53,54]; [55]; [56,57,58,59,60,61]; [62]; [63,64]; [65]
Polymeric micelles	- Spherical, colloidal NP consisting of a hydrophobic core (e.g., PEG) and a hydrophilic corona (e.g., PCL); - Modification of the surface (e.g., antibodies, sugar moieties, peptides) to enhance target specificity	- Activation of the complement system (to a lesser extent than by liposomes)	- Cancer; - Rheumatoid arthritis	[42]; [50,66]
Polymer-drug conjugates	- Macromolecular copolymers (e.g., HPMA) with covalently bound drugs; - pH, redox, enzyme or light sensitive drug release	- HPMA polymer backbone not bio-degradable	- Rheumatoid arthritis; - Spinal cord injury; - Ulcerative colitis	[50,51,67,68,69]; [70]; [71]
Inorganic drug delivery systems	- Inorganic scaffolds or nanoparticles of different size	- Drug load low and stoichiometry difficult to control	- Ocular diseases; - Cancer; - Rheumatoid arthritis	[72]; [73]; [74]
Inorganic–organic hybrid NP	- Composed of equimolar amounts of [ZrO]^2+^ or [GdO]^2+^ and [*R_function_*OPO_3_]^2−^ or [*R_function_*OSO_3_]^2−^; - High drug load	- Toxic effects of the inorganic component not yet reported	- EAE; - Graft-versushost disease; - Acute lung injury	[75]; Unpublished data; Unpublished data

## Abbreviations

BMP	Betamethasone phosphate
Bud	Budenoside
CNS	Central nervous system
Dex	Dexamethasone
EAE	Experimental autoimmune encephalomyelitis
EPR	Enhanced permeability and retention effect
HPMA	*N*-(2-hydroxypropyl) methacrylamide
GC	Glucocorticoids
GR	Glucocorticoid receptor
GRE	Glucocorticoid response element
IL	Interleukin
IOH-NP	Inorganic-organic hybrid nanoparticles
LPS	Lipopolysaccharide
MP	Methylprednisolone
MRI	Magnetic resonance imaging
MRT	Magnetic resonance tomography
MS	Multiple sclerosis
NP	Nanoparticles
PDN	Prednisolone
PEG	Polyethylene glycol
PCL	Poly ε-caprolactone
RA	Rheumatoid arthritis
TAM	Tumor-associated macrophages
TNF	Tumor necrosis factor
